# High p53 Protein Level Is a Negative Prognostic Marker for Pancreatic Adenocarcinoma

**DOI:** 10.3390/ijms252212307

**Published:** 2024-11-16

**Authors:** Sebastian M. Klein, Maria Bozko, Astrid Toennießen, Dennis Rangno, Przemyslaw Bozko

**Affiliations:** 1 Department of Internal Medicine I, Universitätsklinikum Tübingen, Universität Tübingen, Otfried-Müller-Str. 10, 72076 Tübingen, Germany; 2 M3 Research Institute, University Tübingen, 72076 Tübingen, Germany; 3 Institute of Genetics and Biotechnology, Faculty of Biology, University of Warsaw, 02-106 Warsaw, Poland; 4 Faculty of Medicine, University Tübingen, 72076 Tübingen, Germany

**Keywords:** pancreatic adenocarcinoma, p53, patient survival

## Abstract

Pancreatic adenocarcinoma is one of the most aggressive types of cancer. Among different mechanisms generally believed to be important for the development of cancer, aberrant regulation of the p53 protein is a well-known and common feature for many cancer entities. Our work aims to analyze the impact of p53 deregulation and proteins encoded by p53 target genes on the survival of patients suffering from pancreatic adenocarcinoma. We, therefore, focused on the analysis of the selected collective for the TP53 mutation status, the p53 protein level, their correlation, and possible impacts on the prognosis/survival. We compared and analyzed a set of 123 patients. We have extracted information regarding the TP53 mutation status, p53 protein levels, the level of proteins encoded by prominent p53 target genes, and information on the overall survival. Survival analyses were displayed by Kaplan–Meier plots, using the log-rank test, in order to check for statistical significance. Protein levels were compared using the Mann–Whitney Test. We did not find any statistically significant correlation between the TP53 mutation status and the survival of the patients. Moreover, we have not found any significant correlation between the protein amount of prominent p53 target genes and the patients’ survival. However, we see a significant correlation between the p53 protein level in cancer samples and the overall survival of pancreatic adenocarcinoma patients: patients having tumors with a p53 protein level within the upper quartile of all measured cases show a significantly reduced survival compared to the rest of the patients. Thus, in pancreatic adenocarcinoma, the p53 protein level is a relevant marker for prognosis, and cancers having a high p53 protein amount show a shortened patients’ survival. In contrast, for this cancer entity, the TP53 mutation status or the protein amount of prominent p53 target genes on their own seems not to have a significant impact on survival.

## 1. Introduction

Pancreatic ductal adenocarcinoma (PDAC) is a very aggressive type of cancer with a poor prognosis. During the process of malignant transformation towards full malignancy, the cells undergo several stages, such as precursor lesions (pancreatic intraepithelial neoplasia, mucinous cystic and intraductal papillary mucinous neoplasms) and acinar-ductal metaplasia (ADM) [1]. However, in many cases, patients are diagnosed at a late cancer stage with the consequence of a terrible survival prognosis: after a pancreatic adenocarcinoma is diagnosed, the five-year survival rate is no more than 6% in Europe and the USA [2]. Contributing to this is the fact that for PDAC, early metastasis at the time of diagnosis is quite typical, which makes curative surgery, in many cases, not possible or not effective [3,4,5]. Despite many conventional and targeted therapies being proposed, current treatments still have limited success in the course of the disease [6]. Pancreatic cancer can be described as a highly heterogeneous tumor that contains an average of 63 genetic alterations, of which the majority are point mutations. These alterations can be combined into a core set of 12 molecular processes and pathways that were each genetically altered in 67 to 100% of the tumors [7].

Of all the different genetic alterations that are known to contribute to the development of cancer, the mutation of the gene TP53 is one of the most frequent mutations detected in most of the pancreatic adenocarcinoma cases reviewed by [8]. Discovered more than 40 years ago, the p53 protein has been investigated the most within the family of proteins suppressing oncogenesis. The reason for such an important position is the participation of p53 in nearly all types of cellular stresses by coordinating the cellular response to these stimuli—reviewed by [9,10,11,12]. Today, we know that the p53 protein has several different biochemical activities, making it one of the major barriers to cancer development at both the initiation and progression stages. From a biochemical point of view, p53 functions mainly as a sequence-specific transcription factor (TF). p53 binds to specific DNA sequences within the genome (p53 response elements), which leads to the activation of the transcription of target genes—reviewed by [13]. In addition, p53 can also repress the transcription of different genes, usually mediated by indirect mechanisms—reviewed by [14]. Besides its function as a tumor suppressor, the participation of p53 in many other aspects of mammalian physiology has been discovered—reviewed by [9]. In consideration of these observations, p53 should be regarded not only classically as a “guardian of the genome” but as a “guardian of the cell”. Considering such importance of p53, its activity needs to be tightly regulated. p53 is regulated by many proteins, such as murine double minute 2 (Mdm2)/human double minute 2 (Hdm2), p53-induced RING-H2 (Pirh2), Dicer, ADP-ribosylation factor-binding protein 1 (ARF-BP1), silent information regulator sirtuin 1 (Sirt1), CREB-binding protein (CBP)/E1A-binding protein (p300), and JNK, via post-translational modifications, mostly by ubiquitination and phosphorylation—reviewed by [15,16].

Taking into account the importance of the p53 protein for different aspects of tumor cell biology and numerous aberrations (gene mutations and level of the protein) of p53, different researchers and clinicians have tried for a long time to use the mutation status of TP53 and the amount of p53 protein as a prognostic marker for pancreatic adenocarcinoma. However, the data situation has remained contradictory. A very comprehensive study was performed by the group of Dr. Guo, using material from 1472 patients. Tissue microarray was used to evaluate the p53 expression by immunohistochemistry and was categorized as moderate, high, or negative. The authors found that a negative p53 expression correlated with a worse prognosis in patients with resected Pancreatic Ductal Adenocarcinoma [17]. However, this study has several limitations. Firstly, this study defines complete p53-negative tumors as a result of nonsense and frameshift mutations that generate severe truncations. But in many cases, missense mutations can also lead to the translation of a corrupted and unstable protein that will be detected as a negative expression of p53 in the used approach [18]. Secondly, the authors only applied the widely used antibody of p53 N-terminus (DO-7) in the present study. Thus, tumors with mutations in the N-terminus will be recognized as p53 negative [17]. Looking at additional publications, most of the other published data do not support such conclusions, claiming that the amount of p53 protein could not be used as a predictive marker for patients’ survival [19,20,21,22,23,24,25,26,27,28,29,30].

Such an unclear situation requested new studies using new methods to systematically investigate the correlation between TP53 status and the amount of p53 protein in tumor tissues, on the one hand, and clinical parameters like the survival of patients, on the other hand.

Various researchers prepared a list of the genes controlled by p53—systematically reviewed by [31]. In the cited paper, the authors prepared a hierarchal list of positively regulated genes that are important for both cell cycle control and cell death or survival. For our study, we concentrated on the top 20 positively regulated genes. Since one of the main functions of p53 is to regulate the transcription of different genes that are important for regulating the cell cycle and cell death, researchers typically check the amount of mRNA of these p53 target genes. However, in our opinion, it is more important to check the protein levels—because it is the protein and not mRNA molecules that perform the molecular functions in cancer development and the stress-response of cells to different stimuli, including anticancer therapy. Therefore, we checked the protein expression for the top 20 positively regulated genes targeted by p53. Complete information on the amount of the particular protein and patient survival was available for 3 of the 20 candidate genes.

BAX is on the top of the p53 positively regulated genes. It is a proapoptotic protein controlling the mitochondrial-dependent induction of apoptosis—recently reviewed by [32]. For the purpose of this manuscript, we have performed extensive research on the published literature, looking for a possible correlation between the level of this protein and the prognosis for patients suffering from pancreatic adenocarcinoma. However, the only common observation between different publications was a positive correlation between the BAX protein level and the number of apoptotic cells in pancreatic tumor tissues [33,34,35]. Regarding the predictive value of measuring BAX protein level, the opinions differ. Some researchers indicate that a high level of BAX protein in tumors prolongs patients’ survival [33,35], while others report that the amount of BAX protein is not correlated with the patients’ survival [34,36]. Such a contradiction in the potential use of the amount of BAX protein motivated our team to perform our own comparisons in the same manner as we made for p53.

The CDKN1A protein (also called p21) is important for the maintenance of the genome stability by functioning as a cyclin-dependent kinase inhibitor, leading to the arrest of the cell cycle in the G1-phase upon DNA damage or absence of growth factors—recently reviewed by [37,38]. Since the CDKN1 gene (encoding the p21 protein) is one of the most classical transcription targets for the p53 protein, it was obvious to look for a correlation between p53 and p21, on the one hand, and the clinical outcome, on the other hand. However, the clinical data look quite complicated. Some researchers indicate that a high level of p21 protein in PDAC tissues is correlated with good prognosis [25,39], while other studies indicate the absence of any correlation between the amount of p21 and the median survival [27].

Finally, the TP53-induced glycolysis and apoptosis regulator (TIGAR) regulates glycolysis and promotes DNA damage repair and cell proliferation. Biochemical functions of TIGAR include anti-oxidative stress, reducing inflammation, regulating mitochondrial functionality, and inhibiting apoptosis—systematically reviewed by [40]. Moreover, TIGAR is highly expressed in many types of cancers, including PDAC patient-derived xenografts (PDX) with a wild-type TP53—systematically reviewed by [40]. Contrary to p53 itself and p53 target genes like BAX or CDKN1/p21, the potential role of TIGAR has been much less investigated so far. For the moment, only one group had been looking for a correlation between the TIGAR protein expression in PDAC patients. The authors showed that the overall survival of PDAC patients with high TIGAR expression was significantly shorter than that of patients with low TIGAR expression [41]. However, the number of patients in this study was only 23; thus, it is definitively too early for conclusions, so we decided to perform our own correlation analysis in this study.

Summary: Many researchers and clinicians have tried to correlate the mutation status of the TP53 gene, the amount of the p53 protein, and other molecular markers for pancreatic adenocarcinoma with the survival prognosis—systematically reviewed by [28]. However, because of the different approaches used in different studies, it is difficult to really draw credible conclusions when comparing these studies. Thus, for our study, we extracted data for all the analyzed parameters from the same database and re-evaluated the importance of defects in p53 and the protein expression of the most prominent p53 target genes for the prognosis of pancreatic adenocarcinoma.

## 2. Results

### 2.1. Lesson 1: TP53 Is Mutated in the Majority of Pancreatic Adenocarcinomas, Which Influences the p53 Protein Amount Depending on the Type of Mutation

**Figure 1:**
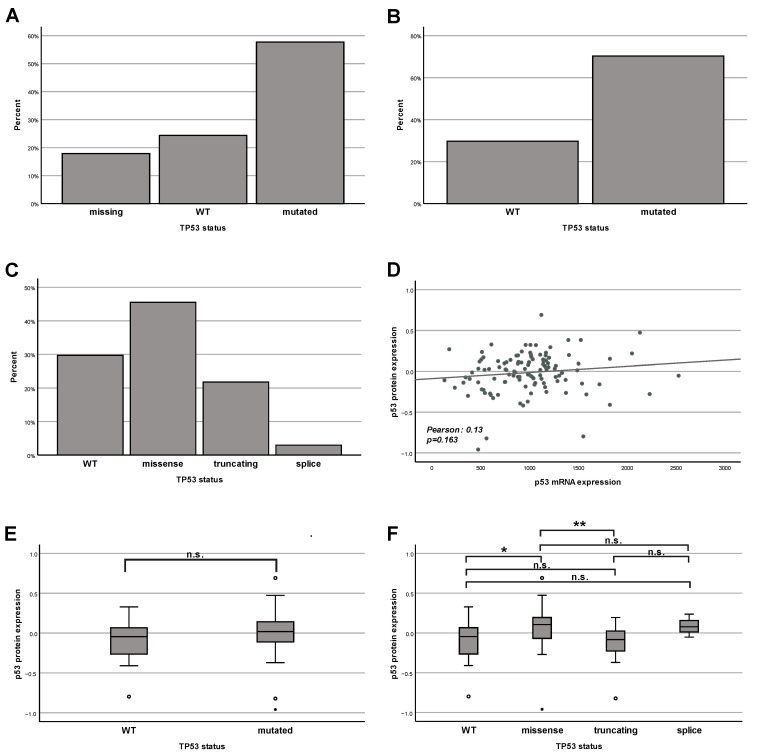
**TP53 is mutated in the majority of pancreatic adenocarcinomas, which influences the p53 protein amount depending on the type of mutation**. (**A**) Frequency of TP53 mutation in pancreatic tumors—all analyzed samples. (**B**) Frequency of TP53 mutation in pancreatic tumors—only samples with available information on TP53 status. (**C**) Frequency of different types of TP53 mutation in pancreatic tumors—only samples with available information on TP53 status. (**D**) Correlation between the p53 mRNA level (RNA Seq V2 RSEM) and the p53 protein level (RPPA, relative values). (**E**) Comparison of the p53 protein level (RPPA, relative values) in pancreatic tumors without and with mutation in TP53. (**F**) Comparison of the p53 protein amount (RPPA, relative values) in pancreatic tumors without and with mutation in TP53 divided into the different mutation types.

First, we analyzed the mutation status of TP53 in our selected cohort of 123 patients. Unfortunately, information on the mutation status was only available for 101 patients (approx. 82% of the total number of patients; Figure 1A). Because the cohort of patients with the known status of TP53 still represents the majority of our analyzed group, we decided to continue with our analysis for this subpopulation at this point. If focusing only on this group (patients with determined status of TP53), the proportion is the following: wild type (WT) 29.7%; mutated 70.3% (Figure 1B). This observation fits perfectly with the generally accepted point of view that the majority of pancreatic tumors show a mutation in TP53—systematically reviewed by [8]—and supports the assumption that the analyzed data set is representative and suitable for making new conclusions. Next, we compared the proportion of different types of TP53 mutation. We found that the most prevalent type of mutations are missense mutations (37.4% of all cases), while truncating mutations are fewer (17.9%), and splice mutations are almost negligible (2.4%) (Figure 1C). These observations correspond with previous comparisons made by other scientists claiming that the majority of mutations of TP53 in pancreatic cancer (approx. ^2^/_3_) are missense mutations, followed by truncating mutations (approx. ^1^/_3_) [42]. We have also checked for the correlation between the amounts of mRNA encoding for p53 and its protein level. We clearly show the absence (*p* = 0.163) of any statistically significant correlation (Figure 1D). Next, we compared the amount of wild-type and mutated p53 protein in a cohort of 101 patients. We have not observed a real statistically significant difference between both groups; however, we see a clear tendency (*p* = 0.09) that the p53 protein level in tissues from patients with tumors carrying a mutated version of TP53 is increased compared to tumors without a mutation in TP53 (Figure 1E). Similar observations were made in varying tumor models in different laboratories—reviewed by [43,44,45,46] and cited articles. To evaluate this observation in more detail, we have asked the question of how the amount of p53 protein depended on the type of TP53 mutation. We observed that the subgroup of tumors with a missense mutation, which was the most frequent (Figure 1C) mutation type, was also the subgroup with the highest amount of p53 protein (Figure 1F) with a statistically significant difference compared to WT (U = 449.00, Z = −2.561, *p* = 0.01) and truncated mutations of TP53 gene (U = 265.00, Z = −3.159, *p* = 0.002). This, again, confirms the importance of missense mutations in pancreatic carcinogenesis [42].

### 2.2. Lesson 2: In Pancreatic Adenocarcinoma, the Mutation Status of TP53 Is Not Suitable to Be a Main Marker for Prognosis on Its Own

**Figure 2:**
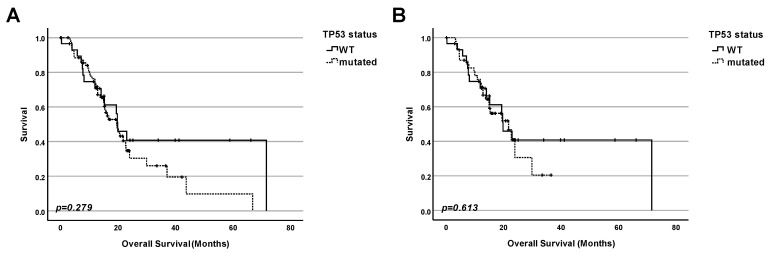
**TP53 status of pancreatic adenocarcinomas has no significant impact on survival**. (**A**) Survival analysis comparing patients carrying tumors with a mutation of the TP53 gene with patients having tumors without a mutation in TP53. (**B**) Survival analysis comparing patients with tumors with a WT TP53 status with patients carrying missense mutation of TP53 gene in the tumor tissue.

For a long time, the prognostic importance of determining the TP53 mutation status itself, as well as the exact type of TP53 gene mutation, has remained absolutely unclear in the case of pancreatic carcinoma—systematically reviewed by [28]. One of the main reasons for this are differences in the methods used to assess the TP53 mutation status and the classification of different TP53 mutations into functionally relevant groups. In our study, we used two types of analysis for our collective of 101 patients:

(1) Initially, we compared the survival of the group of patients with WT TP53 tumors and the group of patients carrying TP53 mutated tumors. We did not find any statistically significant (*p* = 0.279) correlation between the TP53 status and the overall survival. We only observed a very minor tendency towards a decreased life span for patients with a mutation in TP53 (Figure 2A).

As we have previously mentioned, in most types of tumors, missense mutations represent the most frequent fraction within the spectrum of different types of TP53 mutations, reviewed by [44,46] and cited articles and also in our own analysis of pancreatic cancer patients (Figure 1C). In parallel, pancreatic cancer tissues with this kind of mutation show the most prominent p53 protein levels (Figure 1E);

(2) For these reasons, we compared the survival of patients without mutation of the TP53 gene in tumor tissues with patients having a tumor with a missense mutation of TP53. However, in this type of analysis, we also did not find any statistically significant correlation or any tendency (*p* = 0.613) between TP53 status and patient survival (Figure 2B).

### 2.3. Lesson 3: In Pancreatic Adenocarcinoma, High Amount of Total p53 Protein Is a Very Strong Indicator for Bad Prognosis

**Figure 3:**
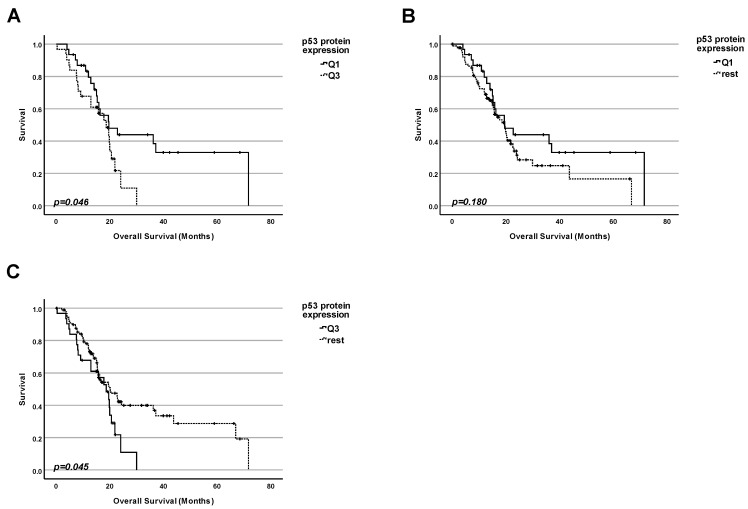
**High p53 protein amounts in pancreatic adenocarcinomas, in general, have a significant negative impact on patients’ survival**. (**A**) Survival analysis comparing the survival of patients having the lowest 25% of p53 protein levels in the cancer tissue (Q1) with the survival of the patients with the highest 25% of p53 protein levels (Q3). (**B**) Survival analysis comparing the survival of patients having the lowest 25% of p53 protein levels in the cancer tissue (Q1) with the survival of the rest of patients. (**C**) Survival analysis comparing the survival of patients having the highest 25% of p53 protein levels in the cancer tissue (Q3) with the survival of the rest of patients.

Following this, we compared if the level of total p53 protein in tumors could be correlated with the overall survival (Figure 3). Data from 123 patients were included in this analysis. We performed three kinds of comparisons by dividing the collective into groups according to the p53 protein level:

1. Firstly, we performed a comparison of the survival for patients with the highest 25% of p53 protein levels in tumors and those with the 25% of the lowest amounts (Figure 3A);

2. Secondly, we performed a comparison of the survival for patients having the lowest 25% of p53 protein levels in tumors and the rest of the patients (Figure 3B);

3. Thirdly, we performed a comparison of the survival for patients having the highest 25% of p53 protein levels in tumors and the rest of the patients (Figure 3C).

While there is no significant difference in the overall survival when comparing patients that have tumors with the lowest 25% of p53 protein levels versus the rest of the patients, we found a significant difference in the survival rate whenever using the subgroup of patients that have tumors with the highest 25% of p53 protein levels. This group shows a significantly reduced survival compared to either the group of patients with tumors with the lowest 25% of p53 protein levels or the rest of the patients in general: *p* = 0.046 for comparison 1 and *p* = 0.045 for comparison 3.

### 2.4. Lesson 4: In Pancreatic Adenocarcinoma, the Protein Level of BAX Cannot Be Used as a Marker for Prognosis Regardless of TP53 Status

**Figure 4:**
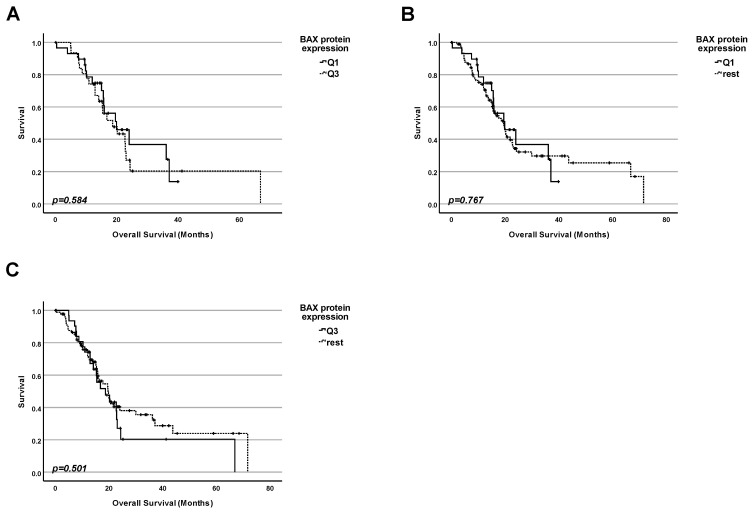
**Protein level of BAX protein cannot be used as a marker for prognosis for pancreatic adenocarcinoma patients, in general.** (**A**) Survival analysis comparing the survival of patients having the lowest 25% of BAX protein levels in the cancer tissue (Q1) with the survival of the patients with the highest 25% of BAX protein levels (Q3). (**B**) Survival analysis comparing the survival of patients having the lowest 25% of BAX protein levels in the cancer tissue (Q1) with the survival of the rest of patients. (**C**) Survival analysis comparing the survival of patients having the highest 25% of BAX protein levels in the cancer tissue (Q3) with the survival of the rest of patients.

Since we have shown that rather than the total amount of the p53 protein and not the mutation status of the TP53 gene is correlated with the patients’ survival, we investigated whether the amount of BAX, a protein well-known to be encoded by one of the p53 target genes, in tumors, could be correlated with the patients’ survival regardless of the status of TP53 (Figure 4). For this study, information from 123 patients was available and used. We performed three types of comparisons by dividing the collective into groups according to the BAX protein level:

1. Firstly, we performed a comparison of the survival for patients with the highest 25% of BAX protein levels in tumors and those with the 25% of the lowest amounts (Figure 4A). No significant difference in survival between the patients in both groups was found (*p* = 0.584);

2. Secondly, we performed a comparison of the survival for patients having the lowest 25% of BAX protein levels in tumors and the rest of the patients (Figure 4B). No significant difference in survival between the patients in both groups was found (*p* = 0.767);

3. Finally, we performed a comparison of the survival for patients having the highest 25% of BAX protein levels in tumors and the rest of the patients (Figure 4C). No significant difference in survival between the patients in both groups was found (*p* = 0.501).

### 2.5. Lesson 5: In Pancreatic Adenocarcinoma, the Protein Level of BAX Cannot Be Used as a Marker for Prognosis Even for Patients with WT TP53 Status

Since the BAX gene is a famous transcription target for p53, it was logical to check if the amount of BAX protein was correlated with survival exclusively for the subpopulation of patients with WT TP53.

**Figure 5:**
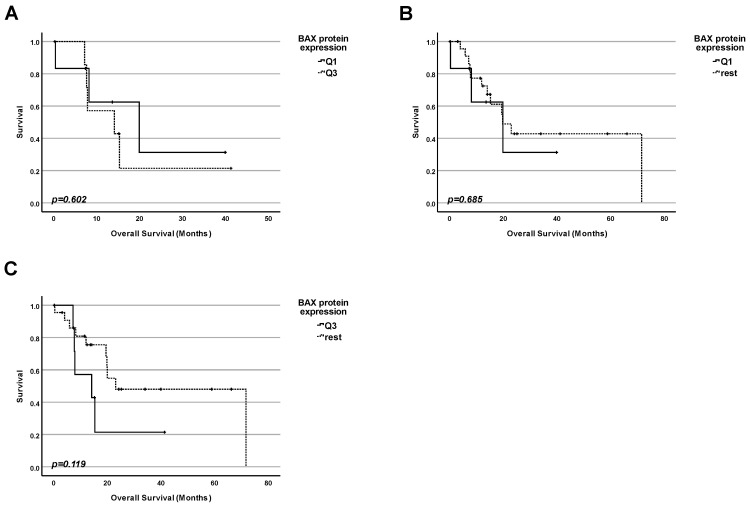
**Protein level of BAX cannot be used as a marker for prognosis for pancreatic adenocarcinoma patients carrying a TP53 WT tumor**. (**A**) Survival analysis comparing the survival of patients having the lowest 25% of BAX protein levels in the cancer tissue (Q1) with the survival of the patients with the highest 25% of BAX protein levels (Q3). (**B**) Survival analysis comparing the survival of patients having the lowest 25% of BAX protein levels in the cancer tissue (Q1) with the survival of the rest of patients. (**C**) Survival analysis comparing the survival of patients having the highest 25% of BAX protein levels in the cancer tissue (Q3) with the survival of the rest of patients.

We investigated whether the protein level of BAX in tumors could be correlated with the survival of patients with WT TP53 (Figure 5). Based on the availability of data, we were able to include datasets of 30 patients for this study. We performed three kinds of analyses by dividing the collective into groups according to the BAX protein level:

1. Firstly, we performed a comparison of the survival for patients with the highest 25% of BAX protein levels in tumors and those with the 25% of the lowest amounts (Figure 5A). No significant difference in survival between the patients in both groups was found (*p* = 0.602);

2. Secondly, we performed a comparison of the survival for patients having the lowest 25% of BAX protein levels in tumors and the rest of the patients (Figure 5B). No significant difference in survival between the patients in both groups was found (*p* = 0.685);

3. Finally, we performed a comparison of the survival for patients having the highest 25% of BAX protein levels in tumors and the rest of the patients (Figure 5C). No significant difference in survival between the patients in both groups was found (*p* = 0.119).

We clearly show that there is no significant correlation between the amount of BAX protein and patients’ survival in pancreatic tumors regardless of the TP53 status.

### 2.6. Lesson 6: In Pancreatic Adenocarcinoma, the Protein Level of p21 (CDKN1A) Cannot Be Used as a Marker for Prognosis Regardless of TP53 Status

**Figure 6:**
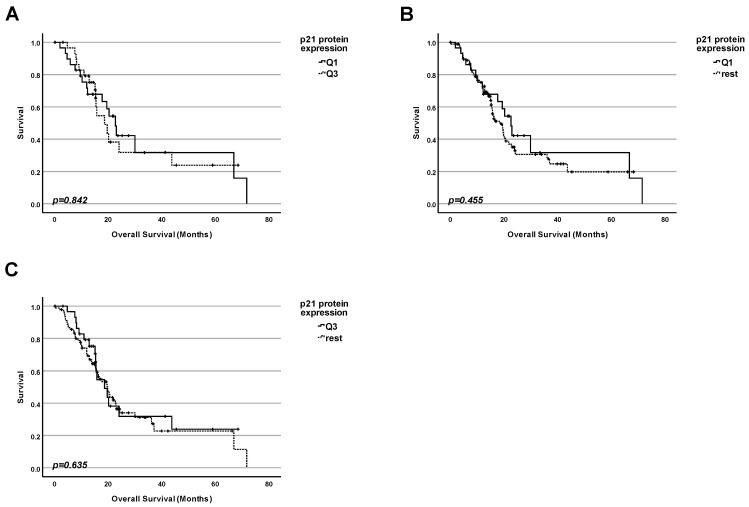
**Protein level of p21 (CDKN1A) cannot be used as a marker for prognosis for pancreatic adenocarcinoma patients, in general.** (**A**) Survival analysis comparing the survival of patients having the lowest 25% of p21 protein levels in the cancer tissue (Q1) with the survival of the patients with the highest 25% of p21 protein levels (Q3). (**B**) Survival analysis comparing the survival of patients having the lowest 25% of p21 protein levels in the cancer tissue (Q1) with the survival of the rest of patients. (**C**) Survival analysis comparing the survival of patients having the highest 25% of p21 protein levels in the cancer tissue (Q3) with the survival of the rest of patients.

Since the total amount of the p53 protein and not the mutation status of the TP53 gene is correlated with the patient’s survival (Figure 2 and Figure 3), we investigated whether the amount of p21 protein, another very prominent protein known to be encoded by one of the p53 target genes, in tumors, could be correlated with the patients’ survival in both WT and mutated TP53 (Figure 6). Data from 123 patients were included in this study. We performed three types of analyses by dividing the collective into groups according to the p21 protein level:

1. Firstly, we performed a comparison of the survival for patients with the highest 25% of p21 protein levels in tumors and those with the 25% of the lowest amounts (Figure 6A). No significant difference in survival between the patients in both groups was found (*p* = 0.842);

2. Secondly, we performed a comparison of the survival for patients having the lowest 25% of p21 protein levels in tumors and the rest of the patients (Figure 6B). No significant difference in survival between the patients in both groups was found (*p* = 0.455);

3. Finally, we performed a comparison of the survival for patients having the highest 25% of p21 protein levels in tumors and the rest of the patients (Figure 6C). No significant difference in survival between the patients in both groups was found (*p* = 0.635).

### 2.7. Lesson 7: In Pancreatic Adenocarcinoma, the Amount of p21 (CDKN1A) Protein Cannot Be Used as a Prognostic Factor Even for Patients with WT TP53 Status

Since CDKN1A is the “number one” transcription target for p53, it was logical to also check whether the amount of the p21 protein was correlated with the survival exclusively for the subpopulation of patients without mutation of TP53.

**Figure 7:**
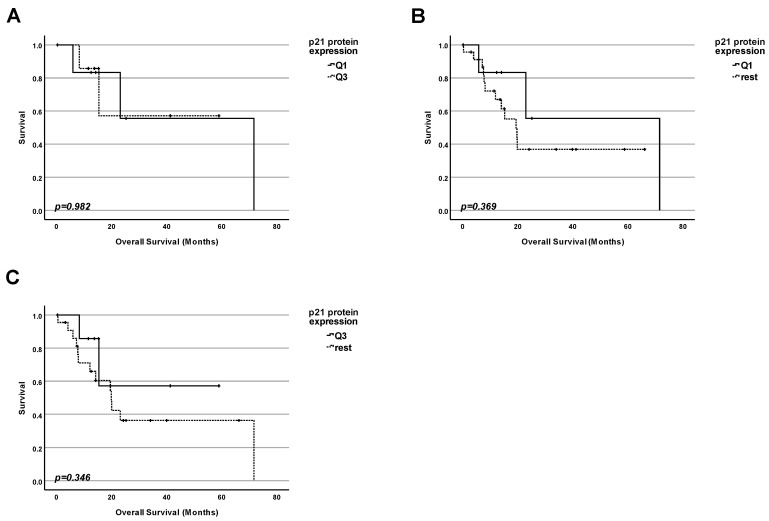
**Protein level of p21 (CDKN1A) cannot be used as a marker for prognosis for pancreatic adenocarcinoma patients carrying a TP53 WT tumor**. (**A**) Survival analysis comparing the survival of patients having the lowest 25% of p21 protein levels in the cancer tissue (Q1) with the survival of the patients with the highest 25% of p21 protein levels (Q3). (**B**) Survival analysis comparing the survival of patients having the lowest 25% of p21 protein levels in the cancer tissue (Q1) with the survival of the rest of patients. (**C**) Survival analysis comparing the survival of patients having the highest 25% of p21 protein levels in the cancer tissue (Q3) with the survival of the rest of patients.

We investigated whether the amount of p21 in tumors could be correlated with the survival of the patients with WT TP53 (Figure 7). Data from 30 patients were included in this study. We performed three kinds of analyses by dividing the collective into groups according to the p21 protein level:

1. Firstly, we performed a comparison of the survival for patients having the highest 25% of p21 protein levels in tumors and those with the 25% of the lowest amounts (Figure 7A). No significant difference in survival between the patients in both groups was found (*p* = 0.982);

2. Secondly, we performed a comparison of the survival for patients having the lowest 25% of p21 protein levels in tumors and the rest of the patients (Figure 7B). No significant difference in survival between the patients in both groups was found (*p* = 0.369);

3. Finally, we performed a comparison of the survival for patients with the highest 25% of p21 protein levels in tumors and the rest of the patients (Figure 7C). No significant difference in survival between the patients in both groups was found (*p* = 0.346).

To summarize, we did not find any significant correlation between the protein level of p21 and the overall survival for tumors with or without mutation of TP53.

### 2.8. Lesson 8: In Pancreatic Adenocarcinoma, the Protein Level of TIGAR Cannot Be Used as a Marker for Prognosis for Patients with and Without Mutation of TP53

**Figure 8:**
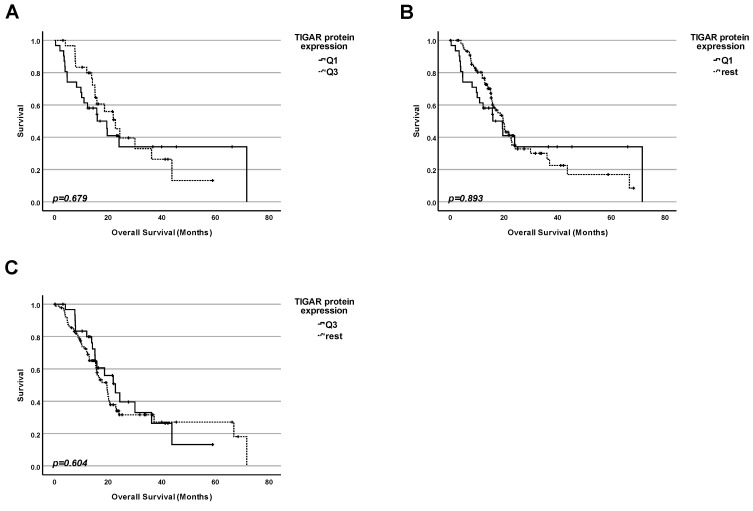
**Protein level of TIGAR cannot be used as a marker for prognosis for pancreatic adenocarcinoma, in general.** (**A**) Survival analysis comparing the survival of patients having the lowest 25% of TIGAR protein levels in the cancer tissue (Q1) with the survival of the patients with the highest 25% of TIGAR protein levels (Q3). (**B**) Survival analysis comparing the survival of patients having the lowest 25% of TIGAR protein levels in the cancer tissue (Q1) with the survival of the rest of patients. (**C**) Survival analysis comparing the survival of patients having the highest 25% of TIGAR protein levels in the cancer tissue (Q3) with the survival of the rest of patients.

Next, we investigated whether the protein level of TIGAR in tumors could be correlated with overall survival regardless of the status of TP53 (Figure 8). Data from 123 patients were included in this study. We performed three kinds of analyses by dividing the collective into groups according to the TIGAR protein level:

1. Firstly, we performed a comparison of the survival for patients with the highest 25% of TIGAR protein levels in tumors and those with the 25% of the lowest amounts (Figure 8A). No significant difference in survival between the patients in both groups was found (*p* = 0.679);

2. Secondly, we performed a comparison of the survival for patients having the lowest 25% of TIGAR protein levels in tumors and the rest of the patients (Figure 8B). No significant difference in survival between the patients in both groups was found (*p* = 0.893);

3. Finally, we performed a comparison of the survival for patients having the highest 25% of TIGAR protein levels in tumors and the rest of the patients (Figure 8C). No significant difference in survival between the patients in both groups was found (*p* = 0.604).

### 2.9. Lesson 9: In Pancreatic Adenocarcinoma, the Protein Level of TIGAR Cannot Be Used as a Marker for Prognosis Even for Patients with WT TP53 Status

Since the TIGAR gene is another transcription target for p53, it was logical to check whether the amount of the TIGAR protein was correlated with survival exclusively for the subpopulation of patients with WT TP53.

**Figure 9:**
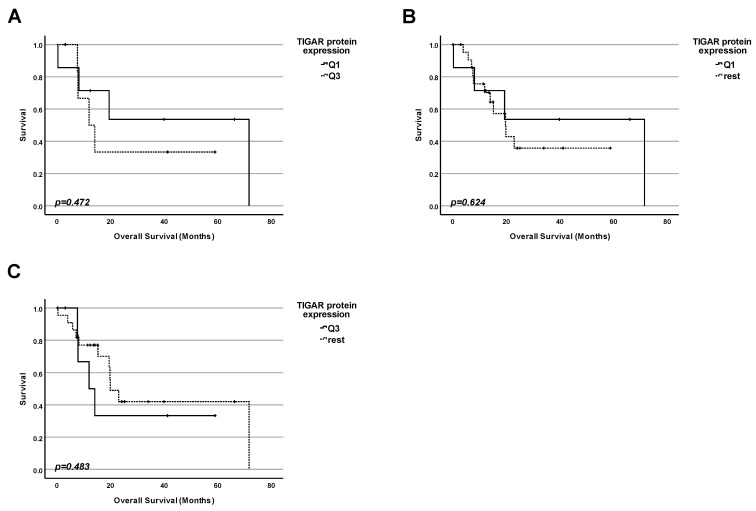
**Protein level of TIGAR cannot be used as a prognostic marker for pancreatic adenocarcinoma patients carrying a TP53 WT tumor.** (**A**) Survival analysis comparing the survival of patients having the lowest 25% of TIGAR protein levels in the cancer tissue (Q1) with the survival of the patients with the highest 25% of TIGAR protein levels (Q3). (**B**) Survival analysis comparing the survival of patients having the lowest 25% of TIGAR protein levels in the cancer tissue (Q1) with the survival of the rest of patients. (**C**) Survival analysis comparing the survival of patients having the highest 25% of TIGAR protein levels in the cancer tissue (Q3) with the survival of the rest of patients.

We investigated whether the amount of TIGAR in tumors could be correlated with the survival of patients with WT TP53 (Figure 9). Data from 30 patients were included in this study. We performed three types of analyses by dividing the collective into groups according to the TIGAR protein level:

1. Firstly, we performed a comparison of the survival for patients with the highest 25% of TIGAR protein levels in tumors and those with the 25% of the lowest amounts (Figure 9A). No significant difference in survival between the patients in both groups was found (*p* = 0.472);

2. Secondly, we performed a comparison of the survival for patients having the lowest 25% of TIGAR protein levels in tumors and the rest of the patients (Figure 9B). No significant difference in survival between the patients in both groups was found (*p* = 0.624);

3. Finally, we performed a comparison of the survival for patients having the highest 25% of TIGAR protein levels in tumors and the rest of the patients (Figure 9C). No significant difference in survival between the patients in both groups was found (*p* = 0.483).

TIGAR is, without question, an important player in tumor development. However, we did not find any significant correlation between the protein level of TIGAR and patients’ survival in tumors regardless of the TP53 status.

## 3. Discussion

The product of the TP53 gene—the tumor suppressor protein p53—belongs to the first league of players both in the development of cancer and in antitumor therapy. When screening The National Library of Medicine (NLM) database—the world’s largest biomedical library—we obtained about 119,000 hits. Most of the other genes or proteins that are also known to be important in the field of cancer reach much lower scores. The p53 protein is involved in the response of the cell to a broad spectrum of stimuli and stressors—systematically reviewed [9,10,11,12]. This reasonably led to the aim of trying to correlate different aspects of the p53 regulation and patients’ survival. This approach has become feasible by the creation of different databases containing both molecular data (for example, mutation status of particular genes and the amounts of particular mRNA and proteins), on the one hand, and survival statistics for a large number of patients, on the other hand. As we have already mentioned in the introduction, numerous researchers and clinicians have tried to use the mutation status of TP53 and p53 protein amounts as prognostic markers for pancreatic adenocarcinoma for a long time. However, the conclusions are strongly different [17,18,19,20,21,22,23,24,25,26,27,28,29,30,47]. Potential reasons include different experimental approaches and ways of calculations. There is a similar problem with the TP53 target gene products, namely, the BAX protein [33,34,35,36,48,49] or p21 [25,27,39,50,51]. Regarding correlations between the TIGAR protein amount and the survival of PDAC patients, only one study has been published so far [41]. In this situation, new studies using new experimental approaches to analyze the correlation between molecular data, including the TP53 status, the p53 protein amount, and the number of protein products of TP53 target genes (BAX, p21, TIGAR), on the one hand, and clinical parameters like survival of patients, on the other hand, are urgently requested. For this reason, we have used data from the TCGA research network that included extensive protein data generated by using the Reverse-Phase Protein Array. The strong point of this method is based on the following principle: reverse-phase protein array (RPPA) is a high-throughput antibody-based targeted proteomics platform that can quantify hundreds of proteins in thousands of samples derived from cell lysates, tissues, or fluids. Protein samples are robotically arrayed on nitrocellulose-coated glass slides. Each slide is probed with a specific antibody that can detect many parameters like levels of total protein expression or post-translational modifications [52]. This leads to a more precise quantification of the results. For our own analysis, we chose the cBioPortal (https://www.cbioportal.org, accessed on 23 November 2023) database [53,54,55] because of its huge collection of datasets and their accessibility, as explained in more detail in the next section [56]. First, we have compared the proportion of both mutated and WT forms of TP53, on the one hand, and the frequency of different types of mutations, on the other hand. In agreement with previously published papers, we found that most pancreatic cancer patients had a mutation of TP53 [8], and that the most frequent type of TP53 mutations were missense mutations [42]. For this reason, we have tried to clarify the importance of this marker for prognosis (Figure 2), showing that the mutation status of TP53 on its own could not be used for prognostic conclusions. However, we found that in the case of pancreatic adenocarcinomas, a high amount of total p53 protein was a very strong indicator of bad prognosis. This indicates that intuitively logical results (coming from cell culture experiments) are not always reproducible in real life. For example, Phan and colleagues showed that downregulation of WT p53 expression was associated with aggressive tumor phenotypes and, presumably, poor prognosis [57], which we were not able to confirm in our analysis of clinical data. Since we and others have shown that, in most cases, TP53 was mutated in pancreatic adenocarcinomas ((Figure 1) [8]), and since, in many cases, the mutated form of p53 had a tendency to accumulate in tumor cells (and, thus, became the attractive target for therapy)—systematically reviewed [43,44,45,46]—we checked for possible correlations between the number of proteins encoded by p53 target genes and survival in the next step. We performed analyses for both patients regardless of the TP53 status and separately for patients with WT TP53. Typically, researchers have checked the amount of mRNA of p53 target genes. However, in our opinion, it is more important to check the protein levels because proteins, and not mRNA, mainly perform particular functions in cancer development and in the response of cells to different types of stress and anticancer therapy. Thus, we checked the protein expression for some genes from the top of positively regulated genes that are targeted by p53. However, no significant correlation with the patients’ survival was found—neither for BAX nor CDKN1p21, nor TIGAR (Figure 4, Figure 5, Figure 6, Figure 7, Figure 8 and Figure 9). In a nutshell, our work clearly shows that in pancreatic adenocarcinoma, the p53 protein level is a relevant marker for prognosis, and cancers having a high p53 protein amount show a shortened patients’ survival. In contrast, for this cancer entity, the TP53 mutation status on its own, as well as the protein level of prominent p53-target genes, seem not to have a significant impact on survival. While we believe that our study can provide substantial new aspects for the stratification of the patients’ prognoses due to the methodological advantages mentioned earlier, this study has its limitations. Our approach was to achieve the most reliable results by trying to keep the analyzed collective of patients as big as possible. We, therefore, performed our analyses for the whole group of patients suffering from PDAC. Of course, as mentioned in Table 1, this collective is not homogenous but diverse in terms of gender, tumor stage, etc. All these parameters might have an impact on the survival and the prognosis. However, dividing our collective of patients into different subgroups based on these clinical parameters would have decreased the number of analyzed patients per group significantly and also reduced the representativeness. By looking at the whole collective of patients, we obtain statistical information across all potential subgroups, which is also a strong point. In the future, it will be worthwhile to split the analyzed cohort into different subgroups based on their clinical characteristics as soon as new data for a much larger collective become available. Then, it would also be worthwhile to reassess the subgroups, whose statistical analysis has been limited to date due to a high proportion of censored data.

## 4. Material and Methods

We extracted data from The Cancer Genome Atlas (TCGA) database provided by GDAC Firehose [56] and finally accessed via cBioPortal (https://www.cbioportal.org, accessed on 23 November 2023) [53,54,55] in the same manner as previously performed and published by our team for ovarian cancer [58]. Data included molecular parameters taken from cancer tissue as well as patient clinical information. In brief, we selected a collective of 123 patients based on the criteria of available information on both the p53 protein level and the overall survival. For the selection of particular p53 target genes for our analysis, we have screened the top 20 positively regulated genes, as reviewed by Fischer [31], for the availability of information regarding protein level and patients’ survival and found three proteins (BAX, p21, and TIGAR) fulfilling these criteria. Data were accessed via cBioPortal and downloaded for our own analyses from either the “plot” section of the website for the gene/protein-related values or from the “clinical summary” section for the clinical data. For the preparation of the data, MS Excel was used. Final statistical analyses were performed in IBM SPSS. The Pearson correlation was used to analyze the relation between the p53 mRNA level and its protein level. Survival analyses were visualized by Kaplan–Meier plots with (+) indicating censored data. The log-rank test was used to check for statistical significance. The datasets were divided into subgroups based on the level of the respective protein, using the quartiles for classification. The fraction below the first quartile was called Q1 (lowest 25%), and the fraction beyond the third quartile, Q3 (highest 25%). For the statistical analysis comparing the protein levels of our selected subgroups, we used the Mann–Whitney Test. *p*-values < 0.05 were defined as statistically significant (* = *p* < 0.05; ** = *p* < 0.01), whereas *p*-values ≥ 0.05 were defined as not significant (ns). For better readability and transparency of the results, we have organized the results section in the form of several lessons, allowing for a similar structure for all of them. Values for protein levels, genetic status, and other molecular markers were taken from tumor tissue unless stated otherwise.

## 5. Conclusions

The study of the role of p53 in both the carcinogenesis process and antitumor therapy is, without question, one of the most important issues both in tumorigenesis and anticancer treatment. However, in pancreatic adenocarcinoma, the determination of TP53 status does not seem to have a prognostic relevance on its own. Moreover, we have analyzed accessible data regarding the protein expression for three of the top 20 transcription targets of p53. For any of the analyzed proteins (BAX, p21, TIGAR), we have not found any correlation between the amount of the protein and patients’ survival. On the contrary, a high amount of p53 protein in tumor tissues is significantly correlated with a worse prognosis, and so the determination of the p53 protein amount in biopsies from patients with pancreatic tumors would be very useful for prognostic conclusions.

## Figures and Tables

**Table 1:** **Clinical characteristics of the analyzed collective of patients suffering from PDAC.** Calculation based on available data for the respective population as indicated if not stated otherwise. (^x^) Calculation based on available data for 122 cases.

		Total		TP53 WT Only		TP53 Mut Only	
		Abs.	%	Abs.	%	Abs.	%
**Cases analyzed**		123	-----	30	-----	71	-----
**Sex**	F	57	46.3	14	46.7	34	47.9
	M	66	53.7	16	53.3	37	52.1
**Age at diagnosis [Years]**		65.34	-----	64.73	-----	64.93	-----
**Tumor Stage**	NA	2	1.6	1	3.3	1	1.4
	IA	5	4.1	1	3.3	3	4.2
	IB	8	6.5	2	6.7	4	5.6
	IIA	19	15.4	3	10.0	11	15.5
	IIB	83	67.5	22	73.3	49	69.0
	III	4	3.3	1	3.3	2	2.8
	IV	2	1.6	0	0	1	1.4
Histologic Grade	GX	16	1.6	0	0	0	0
	G1	67	13.0	5	16.7	8	11.3
	G2	38	54.5	19	63.3	38	53.5
	G3	2	30.9	6	20.0	25	35.2
**Fraction Genome altered**		-----	13.87 ^x^	-----	11.26	-----	14.37

## Data Availability

The results shown here are, on the whole, based on data generated by the TCGA Research Network: https://www.cancer.gov/tcga, accessed on 11 November 2024. More information on how the data were accessed can be found in the Materials and Methods section.
